# Antioxidant Potential of Medicinal Plants in the Treatment of Scabies Infestation

**DOI:** 10.3390/molecules29225310

**Published:** 2024-11-11

**Authors:** Marcin Wróblewski, Joanna Wróblewska, Jarosław Nuszkiewicz, Celestyna Mila-Kierzenkowska, Alina Woźniak

**Affiliations:** Department of Medical Biology and Biochemistry, Faculty of Medicine, Ludwik Rydygier Collegium Medicum in Bydgoszcz, Nicolaus Copernicus University in Toruń, 24 Karłowicza St., 85-092 Bydgoszcz, Poland

**Keywords:** acaricidal activity, antioxidants, medicinal plants, natural therapies, oxidative stress, parasite resistance, *Sarcoptes scabiei*, scabies treatment

## Abstract

Oxidative stress, characterized by an overproduction of reactive oxygen species that overwhelm the body’s physiological defense mechanisms, is a key factor in the progression of parasitic diseases in both humans and animals. Scabies, a highly contagious dermatological condition caused by the mite *Sarcoptes scabiei* var. *hominis*, affects millions globally, particularly in developing regions. The infestation leads to severe itching and skin rashes, triggered by allergic reactions to the mites, their eggs, and feces. Conventional scabies treatments typically involve the use of scabicidal agents, which, although effective, are often associated with adverse side effects and the increasing threat of resistance. In light of these limitations, there is growing interest in the use of medicinal plants as alternative therapeutic options. Medicinal plants, rich in bioactive compounds with antioxidant properties, offer a promising, safer, and potentially more effective approach to treatment. This review explores the role of oxidative stress in scabies pathogenesis and highlights how medicinal plants can mitigate this by reducing inflammation and oxidative damage, thereby alleviating symptoms and improving patient outcomes. Through their natural antioxidant potential, these plants may serve as viable alternatives or complementary therapies in the management of scabies, especially in cases where resistance to conventional treatments is emerging.

## 1. Introduction

Scabies is a prevalent dermatological disorder worldwide, with a particularly high incidence of new cases in developing countries. It is a highly contagious ectoparasitic skin infestation caused by the mite *Sarcoptes scabiei* var. *hominis*. Occasionally, *Sarcoptes scabiei* var. *canis* can adapt to humans, leading to scabies outbreaks in immunosuppressed individuals. Scabicides are agents used to treat scabies by targeting and eliminating *S. scabiei* mites and, in some cases, their eggs. The number of available treatment options remains limited. Commonly used scabicides include sulfur compounds, benzyl benzoate, crotamiton (crotonyl-*N*-ethyl-*o*-toluidine), monosulfiram (tetraethyl thiuram monosulfide), malathion (an organophosphate insecticide), lindane (*γ*-benzene hexachloride), and permethrin (a synthetic pyrethroid insecticide) [1,2,3]. Conventional treatment primarily involves topical medications, with oral medications prescribed in certain cases to eradicate the mites. Topical scabicides, such as permethrin cream, are the most commonly used treatments [4]. When topical treatments are insufficient or impractical, oral medications such as ivermectin may be considered [2]. The availability of drugs varies across countries, leading to differences in treatment practices [5]. Despite their effectiveness, conventional treatments can cause side effects, including skin irritation and itching [4]. Resistance to antiscabies agents, such as permethrin and ivermectin, is an increasingly serious concern, and the clinical significance of resistance and the impact of mass treatment programs remain subjects of ongoing research and debate [5]. 

The scabies mite has long been considered a common external parasite that causes only itching. However, recent epidemiological studies indicate that scabies infection is associated with significant morbidity and even mortality, primarily due to secondary bacterial infections [5]. Scabies lesions are often co-infected with *Staphylococcus aureus* and *Streptococcus pyogenes*, as the mites disrupt the skin barrier and secrete molecules that suppress host immune responses, facilitating bacterial colonization [6]. Medicinal plant extracts and bioactive compounds exert anti-inflammatory and immunomodulatory effects by downregulating inflammatory mediators such as interleukin-1β (IL-1β), interleukin-1 (IL-1), interleukin-6 (IL-6), interleukin-17 (IL-17), tumor necrosis factor-α (TNF-α), interferon-gamma (IFN-γ), cyclooxygenase-2, and inducible nitric oxide synthase [7,8,9,10].

The ideal acaricide should act on larvae, nymphs, and adults and have ovicidal, antibacterial, anti-inflammatory, and/or antipruritic properties. This would prevent relapses caused by newly hatched mites, reduce inflammatory skin reactions caused by mite antigens, and prevent the development of pyoderma [2]. Plants possess significant potential for managing and treating wounds and burns due to their antioxidant, anti-inflammatory, and antimicrobial properties [11]. Medicinal plants, as sources of significant bioactive compounds, offer a safe, effective, and patient-friendly natural treatment option for scabies [1,12]. 

Despite advancements in the treatment of scabies, significant challenges remain, particularly concerning the development of resistance to conventional scabicides and the side effects associated with current treatments. The increasing prevalence of resistant *S. scabiei* strains, coupled with limited therapeutic options, underscores the need for alternative treatment strategies that are both effective and safe. A growing body of research suggests that reactive oxygen species (ROS) play a critical role in the pathogenesis of scabies, exacerbating skin damage and delaying healing. However, the potential for medicinal plants, rich in antioxidants and bioactive compounds, to mitigate the effects of ROS during scabies infestation has not been fully explored in clinical settings. Furthermore, there is a lack of comprehensive studies evaluating the efficacy of these plants in directly reducing oxidative stress and inflammation caused by ROS. This review aims to address these gaps by synthesizing current research on the antioxidant properties of medicinal plants and their potential role in treating scabies. By focusing on ROS and oxidative stress as therapeutic targets, this article seeks to provide a rationale for the use of plant-based treatments that could improve patient outcomes and offer safer, more sustainable alternatives to conventional scabicides. Additionally, the review explores how these natural remedies can address both parasitic eradication and the alleviation of secondary symptoms, such as inflammation and ROS-induced damage, thereby offering a holistic approach to scabies treatment. This synthesis of knowledge may help bridge the gap between traditional remedies and modern clinical practices, promoting the integration of antioxidant-rich plants into scabies management strategies.

## 2. *Sarcoptes Scabiei* and Oxidative Stress

When the production of ROS exceeds the capacity of the antioxidant defense system, free radicals begin to interact with endogenous macromolecules. This interaction causes metabolic dysfunction and oxidative damage to key biomolecules such as lipids, DNA, carbohydrates, and proteins. Consequently, these damages lead to pathological changes in tissues, manifesting themselves in various forms that disrupt normal cellular processes and contribute to a number of diseases [13]. Oxidative stress and ROS are pivotal factors in the pathogenesis of many ectoparasitic and skin diseases, contributing significantly to tissue damage [14]. Oxidative stress induced by ROS can lead to various dermatological conditions, such as erythema, sunburn, atopic dermatitis, psoriasis, contact dermatitis, urticaria, and acne vulgaris [15,16,17,18]. The assessment of oxidative stress provides critical insight into the level of tissue damage in the host system and serves as a valuable indicator of the severity of oxidative damage in various allergic and inflammatory skin diseases in humans [14,19]. ROS initiate the overproduction of lipid peroxides, resulting in an oxidative imbalance. This imbalance, marked by elevated free radical production and reduced antioxidant capacity, causes persistent lipid peroxidation. This process is harmful to the skin because it changes the structure and permeability of cell membranes. Lipid peroxidation causes cell damage by inactivating membrane enzymes and receptors, depolymerizing polysaccharides, and inducing protein cross-linking and fragmentation [13,14]. The by-products of lipid peroxidation, such as lipid hydroperoxides, have been associated with parasite invasion. Elevated levels of lipid peroxides cause cell damage by inactivating enzymes and membrane receptors, altering the structure and permeability of the skin, which may lead to skin lesions caused by *S. scabiei* mites [20]. Malondialdehyde (MDA), a common breakdown product of lipid hydroperoxides, is frequently measured as an indicator of lipid peroxidation [13]. In skin diseases, the body deploys a robust antioxidant defense system, comprising superoxide dismutase (SOD), catalase (CAT), glutathione peroxidase (GPX), glutathione (GSH), and the antioxidant vitamins: vitamin C—ascorbic acid, vitamin E—*α*-tocopherol, *γ*-tocopherol, and vitamin A. The effectiveness of these antioxidants is enhanced by their synergistic and cooperative interactions, which involve the sequential degradation of peroxides and free radicals [21,22]. 

Scabies is a parasitic skin disease caused by the mite *S. scabiei*. Prolonged direct contact, such as sexual interaction with an infected person, is typically considered the primary method of transmission of scabies. However, the mite’s ability to seek out new hosts may facilitate indirect transmission through objects including bedding, towels, and clothing. Table 1 provides an overview of scabies prevalence, risk factors, and epidemiological trends across various regions. These mites cause allergic reactions and skin inflammation in the form of papulovesicular lesions, additionally influencing the secretion of cytokines and chemokines from keratinocytes and skin fibroblasts, and disturbing the balance between immune responses developing through the activation of the Th1 and Th2 pathways [23,24]. Scabies protease paralogs, specifically SMIPP-S-D1, are a group of proteins produced by the scabies mite. These proteins belong to the serine protease family, but have undergone significant mutations that render them catalytically inactive. The exact function of these inactivated proteases is not fully understood, but it is hypothesized that they play a role in mite immune evasion, possibly by interacting with and protecting against host immune proteases [25]. The proinflammatory cytokines triggered by *S. scabiei*, such as IL-1, IL-6, TNF-α, and IFN-γ, can result in the overproduction of ROS [23]. ROS are produced during normal metabolic processes, but their production increases during inflammation and disease [24]. Although extensive research has been conducted on the immune responses and inflammatory processes induced by *S. scabiei* var. *hominis* in humans, detailed studies directly linking scabies infestation in humans with the development of oxidative stress remain limited. Most insights into oxidative stress associated with scabies come from animal models, where pigs are considered the most valuable animal model in dermatological research [26]. In their studies, De et al. [24] and Dimri et al. [20] found that the invasion of *S. scabiei* var. *suis* causes significant changes in oxidative stress markers and impairs the antioxidant defense system in both the blood and skin of infected pigs. Clinical samples collected from pigs suffering from sarcoptic mange showed lower activity of antioxidant enzymes such as SOD, GPX, and CAT, as well as reduced levels of the antioxidant GSH, compares to healthy individuals. Additionally, increased levels of MDA, a product of lipid peroxidation, were associated with clinical signs of sarcoptic mange in pigs. De et al. [24] also demonstrated that infected animals exhibited higher levels of oxidative stress and increased cortisol concentrations compared to control animals. Dimri et al. [20] observed decreased concentrations of ascorbic acid in the blood of pigs suffering from sarcoptic mange. *S. scabiei* has also been reported to cause oxidative stress in rabbits [27], camels [21], dogs [13], and goats [14].

**Table 1:** Regional overview of scabies prevalence, contributing risk factors, and epidemiological trends.

Region	Prevalence Rate (%)		Risk Factor	Trends	Ref.
Africa	Schoolchildren	10.81	Limited access to clean water and poor hygiene practices, including infrequent washing of clothing and linens, increase scabies transmission risk.	Persistent in resource-poor settings with limited water access and hygiene education. Increasing risk in overcrowded institutions.	[28,29,30]
	Institutional Settings (Median)	22.5	Higher prevalence in institutions due to close contact and delayed outbreak response.		
	Sub-Saharan Africa	Up to 33	High-density living conditions and low socioeconomic status increase close personal contact.		
	Specific Sub-Saharan Regions	Up to 65	Seasonal factors, particularly rainy seasons, and events like floods or droughts contribute to transmission.		
Americas	Schoolchildren	0.2 to 20	Common in communities with high-density housing, promoting spread of mites through close contact.	Increasing in crowded urban low-income areas.	[30,31,32,33]
	Schools	Up to 30	Infrequent washing and lack of sanitation in schools contribute to outbreaks.		
	Indigenous Populations	Higher prevalence	Poverty and limited access to healthcare increase prevalence, especially in rural and indigenous communities.		
	Immigrant and Refugee Populations	Higher prevalence	Frequent movement and crowded conditions in temporary housing for immigrants and refugees increase scabies risk.		
Asia	Rohingya Refugee Camps (Bangladesh)	66.42	Overcrowding and environmental factors (e.g., dust, pet exposure, winter season).	Rising in crowded refugee camps, with urgent need for public health interventions.	[34,35,36]
	Southeast Asia (Fiji, Solomon Islands, Timor-Leste)	Up to 33.8	Tropical climate and limited hygiene resources increase risk, especially in young children.		
	Japan (RCFs, Hospitals)	Up to 2.1% in RCFs	Close living in care facilities and hospitals drives outbreaks; high contact within aging populations.		
Europe	Healthcare Workers	1–5	High prevalence among healthcare professionals and institutionalized groups due to occupational exposure.	Rising incidence in urban centers, with peaks noted during colder seasons; increased incidence in healthcare settings and institutions like nursing homes due to higher exposure and poor hygiene practices.	[37,38]
	General Population	Up to 0.5	Increased scabies among refugee populations, and those with high contact rates and limited sanitation access.		
Oceania	General Population	36.4 in Fiji; 16-30 in Vanuatu	Overcrowding, tropical climate, and limited healthcare access contribute to high prevalence rates.	Increasing, especially among children and in high-density villages.	[30,39]

Residential Care Facilities—RCFs.

## 3. Plants and Sarcoptes Scabiei Var. Hominis

For thousands of years, plants have been considered safe medicines in the treatment of many diseases in traditional medicinal systems. Natural products with acaricidal properties can be used as alternative methods of treatment of scabies. In traditional medicine, *Achyranthes aspera* (prickly chaff flower, devil’s whip) is used to treat boils, scabies, skin eruptions, and other skin diseases [40]. Extracts from leaves of *Justicia adhatoda* [41], *Artemisia annua* [42], *Artemisia vulgaris* [43], *Ageratum conyzoides* [44], *Cannabis sativa* [40,45], *Jathrofa curcas* [43], *Acalypha indica* [46], *Clitoria ternatea* [47], *Cassia tora* (*Senna tora*) [48], *Lawsonia inermis* [49], *Briedelia scandens* [48,50], *Boerhaavia diffusa* [51], and *Clerodendrum infortunatum* [51] can be applied to the affected skin to combat scabies. Additionally, *Jathrofa curcas* latex mixed with mustard oil or *Acalypha indica* mixed with garlic, lime, and onion can be used to treat scabies [46,52].

In traditional Nepalese medicine, a yellow paste made from the bark of *Berberis asiatica* is used to treat scabies [53]. Similarly, *Emblica officinalis* (synonym *Phyllanthus emblica*) bark powder, boiled with coconut oil, is taken orally as a medicine for scabies [51,54]. *Juglans regia* bark [44] decoctions and bark powders of various *Ficus* species, including *Ficus recemosa* [40], *Ficus bengalensis* [40,52], and *Ficus carica*, have demonstrated scabies-killing activity, highlighting their value in traditional treatment methods.

In South Africa, the sap from the bark of the stem of *Albizia lebbeck* is used medicinally; an infusion prepared from it is used externally to treat diseases such as mycosis, scabies, and ulcers [53,55]. In Nepal, young shoots of *Lyonia ovalifolia* are traditionally used in folk medicine to treat wounds, cuts, burns, and scabies [43,55]. Moreover, calamus oil extracted from the sap of the *Acorus calamus* rhizome is considered effective in the treatment of scabies [43,53].

*Saraca asoca* flower extract is specifically used to treat scabies in children, highlighting its importance in pediatric care [47,56]. Additionally, the juice from the whole plant of *Alternanthera sessilis* [53] and *Euphorbia neriifolia* [57,58,59] is used externally to treat various skin diseases, including scabies. The milky latex of *Calotropis procera* when mixed with salt is another traditional remedy for scabies, as well as for ringworm, boils, and blisters [53].

*Solanum nigrum* [12,49] and *Blumea lacera* [49] extracts have been shown to effectively alleviate the symptoms of scabies. A review article also suggested that sweet peppers (*Capsicum annuum*) can be used to relieve the pain and itching caused by scabies [12]. Mango tree gum (*Mangifera indica*), originating from India and West Africa, is used in dressings for cracked feet and scabies, demonstrating its versatile use in skin care [40]. 

Rosemary (*Rosmarinus officinalis*) oil has proven effective against scabies, and studies have shown that scabies mites can be killed by applying lavender (*Lavandula officinalis*) oil to the skin [12]. *Mentha piperita* (peppermint) is another herb used to treat a variety of skin conditions, including scabies, dermatitis, inflammation, itching, and ringworm [60].

Oral administration of *Leucas aspera* extracts has a scabies-killing effect, while essential oils from *Cedrus deodara* wood are used externally to treat scabies [44]. The fruit oils of the *Aegle marmelos* tree are part of traditional remedies for skin conditions, and both the leaves and seeds of *Strychnos nux-vomica* and *Pongamia pinnata* are effective in treating and relieving the symptoms of scabies [47,51].

In traditional medical systems, the fruits of *Carissa carandas* [61], *Tanacetum cinerariifolium* (*Chrysanthemum cinerariifolium*) [44], and *Begonia picta* [53,62] are used for therapeutic purposes, particularly in the treatment of scabies and other skin diseases, reflecting the wide use of natural remedies in the treatment of these conditions.

Some studies have shown the promising potential of medicinal plants and their active ingredients against *S. scabiei* var. *hominis*. One significant compound, 9-oxo-10,11-dehydroageraphorone (commonly known as euptox A), has significant scabicidal activity. Euptox A, a cadinene sesquiterpene, is the main toxin extracted from *Eupatorium adenophorum*, effectively killing all *S. scabiei* at a concentration of 3–4 mg/mL (*m*/*v*)[63]. *Tecomella undulata*, locally called rohida tree, desert teak, or Marwar teak, has also shown significant potential in scabies control. Methanol extracts from this plant showed 80% and 83% acaricidal activity in vitro and in vivo, respectively, making it a valuable agent in the treatment of scabies [64].

*Heliotropium indicum* leaf paste, known for its medicinal properties, has been used for centuries to treat various skin ailments, including wounds, scabies, or eczema [51]. A study by Siva Saravanan et al. [65] evaluated the acaricidal potential of a herbal preparation called Thelkodukku Chooranam, derived from *H. indicum*, on patients aged 13–60 years infected with *S. scabiei*. The results were encouraging as oral and topical administration of this herbal preparation significantly reduced all signs and symptoms of scabies after one month of treatment. Importantly, no adverse events were reported during the study period, highlighting the safety of this traditional remedy.

Another study by Ali et al. [66] investigated the therapeutic efficacy of a multi-herbal preparation containing *Fumaria indica*, *Swertia chirayita*, *Tephrosia purpurea*, *Sphaeranthus indicus*, and *Ziziphus jujuba* in the treatment of scabies. After just 15 days of treatment, 50% of patients observed complete resolution of itching, 40% healing of itchy lesions, 33% healing of secondary infections, 43% felt relief from burning sensations, and 83% had negative scratches on the skin, indicating the effectiveness of this herbal mixture. Additionally, research on the effect of essential oils on human scabies has shown that mānuka oil (*Leptospermum scoparium*) is moderately effective. In this study, the average mortality time of mites was found to be 30 min (±7.5 min) after direct exposure to a 10% solution of mānuka oil in paraffin oil, highlighting the potential of essential oils in the treatment of scabies [67].

*Tinospora cordifolia*, commonly known as makabuhay, is a well-known medicinal plant commonly found in various traditional medicinal practices. According to a study by Castillo et al. [68], 50% *T. cordifolia* lotion with ethanol showed antiscabies activity comparable to permethrin, a commonly used antiscabies agent. The study noted that using this lotion caused mild side effects such as erythema, itching, and a burning sensation, but no serious risks were reported, making it a potentially safer alternative.

In another study, Fang et al. [69] highlighted the effectiveness of clove oil (*Syzygium aromaticum*) in the treatment of scabies, stating that it was more effective than other essential oils such as palmarosa, geranium, tea tree, lavender, mānuka, bitter orange, eucalyptus, and Japanese cedar. Studies have shown that a 1% solution of clove oil is able to kill all mites in just 20 min, which highlights its strong scabies killing properties.

Further studies investigated the effectiveness of *Eucalyptus globulus* (camphor oil) in the treatment of scabies, particularly zoonotic scabies. Camphor oil when applied with or without glycerin dilution has been found to completely cure the condition at 100%, 75%, and 50% concentrations within 5–10 days. This suggests that camphor oil may be a very effective treatment method [70].

Aromatic trees from the *Verbenaceae* family and *Clerodendrum infortunatum* shrubs are also widely known for their medicinal uses. The study showed that 20% *Lippia multiflora* essential oil had significant scabies killing activity. For optimal healing and prevention, it is recommended to use lippia oil for more than three consecutive days in patients with scabies [71]. Moreover, the study by Oladimeji et al. [72] showed that lippia oil emulsion formulations were more effective and safer than conventional benzyl benzoate emulsion, with an effectiveness of 100% compared to 87% for benzyl benzoate.

Another medicinal plant, *Vitex negundo*, commonly known as chaste tree, has shown noticeable acaricidal activity. Methanol extracts from the dried stems and leaves of this plant have been shown to be effective both in vitro and in vivo, suggesting its potential use in the treatment of scabies in humans. The 30% methanolic extract of *V. negundo* demonstrated 90% acaricidal activity and achieved over 85% effectiveness compared to standard ivermectin treatment, highlighting its potential as a potent alternative in the treatment of scabies [72].

Neem (*Azadirachta indica*) leaf paste has antiseptic properties [49,73]. *Azadirachta indica*, *Blumea lacera*, *Lawsonia inermis,* and *Solanum hannemanii* are used against scabies by folk medicinal practitioners in Bangladesh [49]. Both the seeds and leaves of *Datura stramonium* and *Datura metel* have scabies-killing properties; the seeds are used orally and the leaves are applied topically [73,74]. The seed oils of *Pimpinella anisum* [12] and *Schleichera oleosa* [51,75] are also used to treat skin diseases like scabies.

The *Myrtaceae* family includes many important trees and shrubs that are important in folk medicine, particularly for the production of essential oils. Among them, *Melaleuca alternifolia* and *Syzygium aromaticum* (cloves) are known for their antiscabies properties. Tea tree oil (TTO), obtained from the *M. alternifolia* plant, is a well-known remedy with a complex composition of about 100 ingredients. The main biologically active components of TTO include terpinen-4-ol (T4O), *γ*-terpinene, *α*-terpinene, and monoterpenes such as 1,8-cineole, *p*-cymene, and *α*-pinene. Topical use of TTO is generally associated with a low incidence of side effects, most of which are limited to irritating or allergic reactions to some of its chemical compounds. However, factors such as light, heat, exposure to air, moisture, and long-term storage may affect the stability of TTO, potentially increasing the content of active ingredients such as *p*-cymene [76,77]. The T4O component has been found to inhibit the production of several inflammatory mediators, including tumor necrosis factor (TNF), IL-1, interleukin-8 (IL-8), interleukin-10 (IL-10), and prostaglandin E2, highlighting its potential anti-inflammatory effects [77]. Although there is relatively little research on the use of TTO specifically for the treatment of scabies in humans, research by Walton et al. [78] has shown that a 5% solution of TTO and its active ingredient T4O are effective in reducing the survival time of mites. However, other ingredients such as 1,8-cineole and *α*-terpineol are relatively inactive against scabies. Moreover, TTO shows excellent in vitro activity against *S. scabiei* var. *hominis* when used in combination therapy (combination of 25% benzyl benzoate with 5% TTO). Another study by Liuwan et al. [79] demonstrated the acaricidal effect of TTO formulation (5%, 10% v/w TTO) compared to active permethrin cream (5% w/w permethrin) in treating scabies infestation in children. The results showed that the highest and fastest cure rates were in the 5% TTO cream treatment group. In support of these results, a similar study by Zulkarnain et al. [80] also showed that TTO 5% cream is more effective than permethrin 5% cream in the treatment of scabies in children. Interestingly, the combination of TTO 5% with 5% permethrin was more effective than 5% permethrin ointment alone, although TTO 5% cream alone was found to be the most effective treatment for pediatric scabies. The mechanism underlying the effectiveness of 5% TTO is believed to be blocking the mites’ sodium channels, leading to paralysis and subsequent eradication of the arthropods.

Turmeric (*Curcuma longa*) paste has long been used in traditional medicine to treat scabies, achieving a remarkable cure rate of 97% in just 3 to 15 days of treatment. In the Indian system of medicine, turmeric is often combined with neem leaves to treat various skin eruptions and scabies, reflecting its wide therapeutic potential [72].

In Bangladesh, folk medicinal practitioners use various plants to treat scabies. Among them, *Azadirachta indica* (neem), *Blumea lacera*, *Lawsonia inermis* (henna), *Solanum hannemanii,* and *Inula viscosa* are well appreciated for their effectiveness in fighting scabies [49,81]. *Datura stramonium* and *Datura metel* seeds and leaves also have scabies-killing properties; while the seeds are taken orally, the leaves are applied topically to the affected areas [73,74]. The seed oils of *Pimpinella anisum* [12] and *Schleichera oleosa* [51,75] are also used to treat skin diseases like scabies.

In addition, various other botanical extracts and essential oils have been used to treat scabies. These include palmarosa oil (*Cymbopogon martini*), nutmeg oil (*Myristica fragrans*), ylang-ylang oil (*Cananga odorata*), bitter orange oil (*Citrus aurantium amara*), geranium oil (*Pelargonium asperum*), and Japanese cedar oil (*Cryptomeria japonica*). Moreover, the plant extracts of *Ailanthus altissima* and *Ligularia virgaurea* are also known for their use in the treatment of scabies, which shows the wide range of natural resources used to combat this skin disease [82].

The human skin hosts a diverse array of bacteria, including low-virulence commensal bacteria such as coagulase-negative staphylococci and non-pathogenic *Corynebacterium* spp., alongside pathogenic bacteria like *Staphylococcus aureus* and *Streptococcus pyogenes*. In hospitalized patients, particularly those who have undergone antibiotic therapy, the skin may also be colonized by Gram-negative non-fermentative bacteria or yeasts [83]. Skin infections are frequently caused by fungal species such as *Trichophyton* spp., *Epidermophyton floccosum*, *Malassezia furfur*, *Candida* spp. [84]. A significant issue is that scabies is frequently misdiagnosed and mistreated, as it is often confused with other pruritic conditions such as eczema, impetigo (caused by *S. aureus* and *S. pyogenes*), tinea corporis (ringworm caused by dermatophytes), and psoriasis [64]. The therapeutic potential of medicinal plants lies in their rich diversity of bioactive compounds that contribute to their antimicrobial, antioxidant, and anti-inflammatory properties [85,86]. Among the plants listed in this review article that have antiscabies activity, the neem tree is an important multifunctional species with great potential. Neem shows significant activity against *S. scabiei* var. *hominis*; it is also used for eczema, impetigo, fungal infections, and psoriasis [87,88]. TTO and T4O are broad-spectrum agents, effective against Gram-positive and Gram-negative bacteria, as well as yeasts such as *Candida albicans* in vitro [76]. The broad spectrum of antibacterial activity of TTO forms the basis for its use as an active ingredient in many topical preparations used to treat skin infections. The cumulative effects of the acaricidal, antibacterial, antipruritic, anti-inflammatory, and wound-healing properties of TTO may potentially reduce the risk of scabies infection due to bacterial complications [2,79]. Other plants mentioned in the study may not have such a broad spectrum of activity. Phytochemicals such as phenols, flavonoids, quinones, coumarins, phenolic acids, tannins, terpenes, and alkaloids primarily function as chemical defenses against insects and microorganisms [89]. A summary of selected medicinal plants with antiscabies activity, along with their key compounds and forms of application, is presented in Table 2.

**Table 2:** Review of antiscabies plants, their active compounds, and forms of use.

Commonly Known as	Botanical Name	Therapeutic Action	Form of Application	Ref.
Neem	Azadirachta indica	Shows antiscabies activity, effective with extended use.	5% neem oil cream, applied topically.	[69,90]
Tea tree	Melaleuca alternifolia	Terpinen-4-ol damages mite cell membranes, causing rapid immobilization.	5% essential oil solution, applied topically.	[78]
Turmeric	Curcuma longa	Curcumin has anti-inflammatory and anti-parasitic properties, inhibiting mite growth and reproduction.	Turmeric extract, applied topically.	[12,64]
Rosemary	Rosmarinus officinalis	Camphor and 1,8-cineole demonstrate strong anti-mite activity, reducing survival rates on treated areas.	Essential oil, applied topically.	[91]
Crofton weed	Eupatorium adenophorum	Euptox A exhibits potent acaricidal properties, effectively reducing mite survival.	Purified extract, applied topically.	[63]
Pepper	Capsicum annuum	Capsaicin reduces itching and soothes skin irritation.	Cream or ointment with capsaicin, applied topically.	[92]

## 4. Antioxidant Properties of Medicinal Plants

The skin, our largest organ, serves as a critical barrier between the body and the external environment, protecting against dehydration, pathogens, toxic chemicals, and temperature fluctuations. Due to its constant exposure, the skin is prone to frequent injuries. The healing and regeneration of the skin is a highly intricate process, involving coordinated interactions between various cells, growth factors, and cytokines [93]. Antioxidants play a crucial role in wound healing by protecting tissues from oxidative damage induced by reactive oxygen species (ROS), inhibiting lipid peroxidation, and enhancing the activity of antioxidant enzymes such as superoxide dismutase (SOD). Drugs or bioactive compounds that prevent lipid peroxidation can strengthen collagen fibers, safeguard cells from damage, improve circulation, and promote DNA synthesis, thereby enhancing tissue viability [7]. Medicinal plants may be a source of biologically active compounds potentially used in new preparations for the treatment of skin diseases.

Recently, there has been growing interest in the therapeutic potential of medicinal plants as antioxidants that reduce tissue damage caused by ROS. Antioxidants are molecules that can safely interact with ROS, ending chain reactions before they damage essential molecules [94]. The main groups of antioxidant compounds found in plants include phenolic compounds. The most common phenolic compounds include phenolic acids (such as hydroxybenzoic acid and hydroxycinnamic acid), phenylpropanoids (including phenylpropenes), coumarins (such as herniarin and coumarin), stilbenes, curcuminoids, xanthones, flavonoids (including various subtypes such as flavonols, flavanols, flavones, flavanones, flavonoid glycosides, isoflavones, and anthocyanins), and some cannabinoids and some vitamins (such as vitamin E) [86,95,96,97]. Additionally, lignans, which are also a group of polyphenolic compounds, are common in plants [98,99]. Natural polyphenols can have simple structures such as phenolic acids and flavonoids, or more complex structures such as polymers including lignins, melanins, and tannins. Phenolic compounds play a key role in counteracting oxidative stress, supporting the physiological defense system against ROS. The mechanism of their antioxidant action is strongly dependent on the specific type of phenols found in a given plant extract. Different phenols have varying degrees of effectiveness in neutralizing ROS and protecting against oxidative damage. For example, hydroxybenzoic and hydroxycinnamic acids show significantly higher antioxidant activity in vitro compared to well-known antioxidant vitamins. The presence of various substituents in the aromatic ring of phenolic acids affects their structural stability and the ability to quench radicals, which leads to different antioxidant effects of individual phenolic acids [100]. Phenolic compounds play a key role in stabilizing and protecting cellular structures, including lipids, against oxidative stress [101]. They achieve this by increasing the activity and expression of antioxidant enzymes and neutralizing free radicals or ROS, such as hydroxyl radicals (·OH) and superoxide anions (O_2_^•−^) [102,103]. Additionally, due to their redox properties, they act as reducers, hydrogen donors, metal chelators, and singlet oxygen quenchers [94,95,96,104,105]. Polyphenols also act synergistically with essential vitamins, upregulate key antioxidant enzymes such as SOD, CAT, and GPX, and promote the expression of enzymes involved in glutathione synthesis and phase II drug metabolism by regulating the Nrf2/Keap1 pathway [95]. Flavonoids, a prominent class of polyphenols found in plants, are most intensively studied for their antioxidant and biological activities. They demonstrate strong antioxidant properties in vitro, demonstrating the ability to neutralize a wide spectrum of ROS and reactive nitrogen species (RNS) [101]. Flavonoids inhibit the oxidase and arachidonic acid pathways, reduce the activity of xanthine oxidase (which catalyzes the formation of superoxide radicals), and reduce the activity of membrane NADPH oxidase involved in the production of O_2_^•−^ [95].

Vitamin C, vitamin E, and precursor of vitamin A (*β*-carotene) play a key role in the body as antioxidants, protecting cells against the harmful effects of free radicals. Each of these vitamins has a unique chemical structure and functional group that contributes to their ability to neutralize reactive oxygen species [106].

Terpenoids, the largest group of plant secondary metabolites, are classified based on the number of carbon atoms they contain. These categories include hemiterpenes (C_5_), monoterpenes (C_10_), sesquiterpenes (C_15_), diterpenes (C_20_), triterpenes (C_30_), tetraterpenes or carotenoids (C_40_), and polyterpenes (C_n,_ n > 40) [103,107]. Terpenes perform various important biological functions, such as being essential components of essential oils, plant pigments (like carotenoids), steroid hormones, and precursors to bioactive molecules [108]. Despite their chemical diversity and numerous mechanisms of action, terpenes are assumed to have antioxidant properties [103,108]. These compounds are effective in scavenging ROS and inhibiting lipid peroxidation [109].

Quinones are molecules with high redox activity, and their semiquinone radicals can lead to the formation of ROS, including O_2_^•−^, hydrogen peroxide, and ultimately ·OH [110,111,112]. Quinones act as electron carriers, which allows them to attack mitochondria and restore electron transfer in states of deficiency [110]. There are three main groups of naturally occurring quinones, namely benzoquinones, naphthoquinones, and anthraquinones [111].

Alkaloids are nitrogen-containing compounds with a wide range of pharmacological activities, including antioxidant activity [107]. They can neutralize ROS and inhibit oxidative processes [113].

Saponins are naturally occurring surface-active glycosides commonly found in plants. They consist of a carbohydrate molecule attached to an aglycone, which can be either a triterpenoid or a steroid [114]. The oxidative potential of saponins is manifested primarily through their ability to neutralize ROS, which are highly reactive molecules that can cause oxidative stress in biological systems [98,114].

Arabinogalactans are highly branched, complex heteropolysaccharides consisting mainly of arabinose and galactose residues. These compounds exhibit significant antioxidant activity, which is attributed to their monosaccharide composition and branched structure. It is believed that the antioxidant properties of arabinogalactans result from their ability to scavenge free radicals, reduce metal ions, and inhibit lipid peroxidation [115].

The antioxidant properties of these fatty acids are crucial in protecting cells from oxidative damage, which is linked to various chronic diseases such as cancer and cardiovascular disease. Unsaturated fatty acids, such as linolenic and oleic acid, generally demonstrate higher antioxidant activity due to their multiple double bonds. However, saturated fatty acids like palmitic acid also contribute, albeit to a lesser degree [116].

The various groups of antioxidant compounds found in plants with potential activity against *S. scabiei* var. *hominis* include phenolic compounds, vitamins, alkaloids, terpenoids, saponins, heteropolysaccharides, quinones, and fatty acids (Table 3). The unique composition of medicinal plants influences their antioxidant properties, as their phytochemicals and other bioactive compounds work synergistically to neutralize free radicals and enhance antioxidant enzyme activity. In scabies patients, medicinal plant extracts or bioactive compounds with antioxidant properties may reduce oxidative damage caused by inflammation and scratching associated with the infestation.

The assessment of antioxidant capacity in natural products involves both chemical and cellular assays to capture the full range of antioxidant activity. Chemical methods are commonly used in preliminary screenings due to their cost-effectiveness, high efficiency, and ability to provide comparative values. Examples include the Oxygen Radical Absorption Capacity (ORAC), 2,2-diphenyl-1-picrylhydrazyl (DPPH) scavenging assay, Ferric Reducing Antioxidant Power (FRAP), and newer nanoparticle-based techniques such as the Silver Nanoparticle Antioxidant Capacity (SNPAC) test. While these methods primarily measure the ability of antioxidants to scavenge free radicals or reduce metal ions, they do not fully account for the complexities of biological systems, including factors such as bioavailability and cellular metabolism. To address these limitations, cellular antioxidant activity (CAA) assays are employed, which assess the ability of antioxidants to reduce oxidative stress in living cells by considering factors like membrane permeability and interactions with cellular components [117]. It is crucial to recognize that the antioxidant capacity of natural products is largely determined by the bioavailability of the compound mixture and the synergistic interactions between them, which collectively drive the antioxidant response at the cellular level.

**Table 3:** The antioxidant potential of plant extracts which show significant activity against *S. scabiei* var. *hominis*.

Family	Genus	Species	Major Identified Antioxidant Compounds	Health-Promoting Properties of Plants for Humans	Ref.
*Acanthaceae*	*Justicia*	*adhatoda*	Alkaloids—vasicine, vasicinone, vasicoline. Flavone *C*-glycosides—vicenin-2. Vitamins—*α*-tocopherol, *γ*-tocopherol.	Antioxidant activity. Anti-inflammatory potential. Analgesic. Immunomodulatory effects. Antibacterial activities. * Antifungal activity.	[118,119,120,121,122]
*Acoracee*	*Acorus*	*calamus*	Phenylpropanoids—*α*-asarone, *β*-asarone.	Antioxidant activity. Anti-inflammatory potential. Immunomodulatory effects. Wound-healing effects. Low antibacterial activity. * Antifungal activity.	[123,124,125,126,127]
*Amaranthaceae*	*Alternanthera*	*sessilis*	Carotenoids—astaxanthin Fatty acids—palmitic acid. Flavone *C*-glycosides—vicenin-2. Tannins. Terpenoids—azadirachtin. Vitamins—ascorbic acid.	Antioxidant activity. Anti-inflammatory potential. Analgesic. Wound-healing effects. Antibacterial activities. * Antifungal activity.	[128,129,130,131]
	*Achyranthes*	*aspera*	Flavanols—catechin, epicatechin. Flavonols—quercetin. Hydroxybenzoic acids—gallic acid.	Antioxidant activity. Anti-inflammatory potential. Analgesic. Immunomodulatory effects. Wound-healing effects. Antibacterial activities. Antifungal activity.	[103,132,133,134,135,136,137,138]
*Anacardiaceae*	*Mangifera*	*indica*	Carotenoids. Flavanols—catechin, epicatechin. Flavonols—quercetin. Hydroxybenzoic acids—gallic acid, protocatechuic acid, benzoic acid. Phenolic esters—methyl gallate, propyl gallate, propyl benzoate. Vitamins—ascorbic acid. Xanthonoids—mangiferin.	Antioxidant activity. Anti-inflammatory potential. Analgesic. Immunomodulatory effects. Wound-healing effects. Antibacterial activities. * Antifungal activity.	[139,140,141,142,143,144]
*Annonaceae*	*Cananga*	*odorata*	Phenylpropenes—eugenol. Sesquiterpenes—*β*-caryophyllene, germacrene D, *α*-farnesene. Terpene alcohols—linalool.	Antioxidant activity. Anti-inflammatory potential. Antibacterial activities. * Antifungal activity.	[145,146]
*Apocynaceae*	*Carissa*	*carandas*	Anthocyanins—cyanidin-3-galactoside, delphinidin-3-rutinoside. Terpenoids—carandinol, ursolic acid, betulinic acid. Vitamins—ascorbic acid.	Antioxidant activity. Anti-inflammatory potential. Antibacterial activities. *	[147,148]
	*Calotropis*	*procera*	Hydroxycinnamic acids—caffeic acid, *p*-coumaric acid. Flavones—luteolin. Flavonol glycosides—rutin. Flavanols—catechin. Flavonols—kaempferol.	Antioxidant activity. Anti-inflammatory potential. Immunomodulatory effects. Antibacterial activities.	[103,149,150,151]
*Asteraceae* (*Compositae*)	*Artemisia*	*vulgaris*	Flavones—chrysosplenol D, casticin. Flavonols—quercetin. Phenolic compounds—caffeoylquinic acids. Sterols—*β*-sitosterol. Sesquiterpenes—artemisinin.	Antioxidant activity. Anti-inflammatory potential. Wound-healing effects. Analgesic. Immunomodulatory effects. Antibacterial activities. * Antifungal activity.	[12,93,152,153,154,155,156,157]
		*annua*			
	*Ageratum*	*conyzoides*	Flavonols—quercetin.	Antioxidant activity. Anti-inflammatory potential. Wound-healing effects. Analgesic. Antibacterial activities. *	[7,158,159,160,161]
	*Blumea*	*lacera*	Diterpenes—phytol. Hydroxycinnamic acids—rosmarinic acid. Fatty acids—linolenic acid, oleic acid. Flavonol glycosides—rutin. Flavonols—quercetin, kaempferol. Flavanols—catechin, epicatechin.	Antioxidant activity. Anti-inflammatory potential. Wound-healing effects. Antibacterial activities. *	[162,163]
	*Inula*	*viscosa*	Caffeoylquinic acid. Flavanonols—taxifolin. Flavonols—quercetin.	Antioxidant activity. Anti-inflammatory potential. Wound-healing effects. Antibacterial activities. * Antifungal activity.	[10,81,164,165]
	*Tanacetum*	*cinerariifolium*	Sesquiterpenes	Antioxidant activity. Wound-healing effects.	[166]
*Begoniaceae*	*Begonia*	*picta*	Alkaloids Flavone C-glycosides—vitexin, isovitexin, orientin, isoorientin. Phenolics. Saponins. Tannins.	Antioxidant activity. Antibacterial activities. * Antifungal activity.	[62,167,168]
*Berberidaceae*	*Berberis*	*asiatica*	Hydroxycinnamic acids—caffeic acid, *p*-coumaric acid, chlorogenic acid. Flavonol glycosides—rutin. Hydroxybenzoic acid—vanillic acid	Antioxidant activity. Anti-inflammatory potential. Wound-healing effects. Antibacterial activities. *	[130,169,170,171,172]
*Bignoniaceae*	*Tecomella*	*undulate*	Flavonoids. Phenols.	Antioxidant activity. Anti-inflammatory potential. Antibacterial activities. Antifungal activity.	[173,174,175,176,177]
*Boraginaceae*	*Heliotropium*	*indicum*	Diterpene alcohols—phytol. Monoterpenes—*β*-linalool Sterols—*β*-sitosterol, stigmasterol. Triterpenes—lupeol, *β*-amyrin.	Antioxidant activity. .Anti-inflammatory potential. Analgesic. Wound-healing effects. Antibacterial activities. * Antifungal activity.	[169,178]
*Cannabinaceae*	*Cannabis*	*sativa*	Cannabinoids—cannabidiol, cannabinol, tetrahydrocannabinol. Flavonoids. Terpenoids.	Antioxidant activity. Anti-inflammatory potential. Antibacterial activities. * Antifungal activity.	[83,176,179,180]
*Caprifoliaceae*	*Scabiosa*	*columbaria*	Hydroxybenzoic acids—gallic acid, benzoic acid. Hydroxycinnamic acids—chlorogenic acid, caffeic acid. Flavanols—catechin.	Antioxidant activity. Wound-healing effects. No antibacterial activities. *	[181,182,183]
*Cupressaceae*	*Cryptomeria*	*japonica*	Monoterpenes—terpinen-4-ol. Sesquiterpenes—nezukol, elemol, eudesmol.	Antioxidant activity. Anti-inflammatory potential. Antibacterial activities. *	[184,185]
*Ericaceae*	*Lyonia*	*ovalifolia*	Flavonoids. Phenolics. Tannins. Terpenoids.	Antioxidant activity. Anti-inflammatory potential. Antibacterial activities.	[186]
*Euphorbiaceae*	*Emblica*	*officinalis*	Flavonol glycosides—rutin. Flavonols—quercetin. Hydroxybenzoic acids—gallic acid, ellagic acid.	Antioxidant activity. Anti-inflammatory potential. Immunomodulatory effects. Analgesic. Wound-healing effects. Antibacterial activities. * Antifungal activity.	[187,188,189,190]
	*Euphorbia*	*neriifolia*	Flavonoids. Phenolics. Tannins. Triterpenes—sapogenin, euphol, cycloartenol.	Antioxidant activity. Anti-inflammatory potential. Immunomodulatory effects. Analgesic. Wound-healing effects. Antibacterial activities. * Antifungal activity.	[84,191]
	*Jatropha*	*curcas*	Carotenoids. Flavone *C*-glycosides—vicenin-2, stellarin-2, vitexin, isovitexin. Flavone *O*-glycosides –isorhoifolin, rhoifolin. Phenolics. Vitamins—ascorbic acid.	Antioxidant activity. Anti-inflammatory potential. Wound-healing effects. Immunomodulatory effects. Analgesic. Antibacterial activities. * Antifungal activity.	[83,192,193,194,195,196,197]
	*Acalypha*	*indica*	Hydroxybenzoic acids—gallic acid. Flavonol glycosides—rutin. Flavonoids and related compounds—swietenine, retusoquinone. Porphyrins—coproporphyrin II.	Antioxidant activity. Anti-inflammatory potential. Wound-healing effects. Analgesic. Antibacterial activities. *	[169,198,199,200]
*Fabaceae*	*Pongamia*	*pinnata*	Flavonoids. Furano flavonoids—karanjin, pongapin. Phenolic acids.	Antioxidant activity. Anti-inflammatory potential. Immunomodulatory effects. Wound-healing effects. Antibacterial activities. * Antifungal activity.	[201,202,203]
	*Clitoria*	*ternatea*	Anthocyanins—ternatin. Flavonol glycosides—rutin. Flavanols—epicatechin. Flavonols—quercetin, kaempferol.	Antioxidant activity. Anti-inflammatory potential. Immunomodulatory effects. Wound-healing effects. Antibacterial activities.^*^ Antifungal activity.	[204,205,206,207,208]
	*Albizia*	*lebbeck*	Flavonol glycosides—rutin. Flavones—luteolin. Hydroxybenzoic acids—vanillic acid.	Antioxidant activity (low). Anti-inflammatory potential. Wound-healing effects. Antibacterial activities. * Antifungal activity.	[55,209,210]
	*Cassia*	*tora*	Anthraquinones—chrysophanol, physcion, aurantio-obtusin, chryso-obtusin. Flavonols—quercetin, kaempferol. Glycosides.	Antioxidant activity. Anti-inflammatory potential. Analgesic. Antibacterial activities * (except for *S. aureus*). Antifungal activity.	[211,212,213,214,215]
	*Saraca*	*asoca*	Flavonols—quercetin. Hydroxybenzoic acids—gallic acid, ellagic acid.	Antioxidant activity. Wound-healing effects. Antibacterial activities. *	[216,217]
*Fumariaceae*	*Fumaria*	*indica*	Alkaloids—paprafumine, paprarine, papraline, cryptopine, raddeanine, oxocoptisine.	Antioxidant activity. Anti-inflammatory potential. Analgesic. Wound-healing effects. Antibacterial activities. *	[218,219,220,221]
*Juglandaceae*	*Juglans*	*regia*	Hydroxybenzoic acids—gallic acid, ellagic acid, protocatechuic acid. Hydroxycinnamic acids—*p*-coumaric acid, ferulic acid. Flavonols—quercetin. Naphthoquinones—juglone.	Antioxidant activity. Anti-inflammatory potential. Analgesic. Wound-healing effects. Immunomodulatory effects. Antibacterial activities. * Antifungal activity.	[222,223,224]
*Lamiaceae*	*Lavandula*	*officinalis*	Hydroxycinnamic acids—rosmarinic acid. Flavones—luteolin, apigenin. Flavonols—quercetin. Flavanones—naringenin. Monoterpenes—linalool, linalyl acetate.	Antioxidant activity. Anti-inflammatory potential. Analgesic. Wound-healing effects. Antibacterial activities. * Antifungal activity.	[83,225,226,227]
	*Mentha*	*piperita*	Hydroxycinnamic acids—rosmarinic acid. Flavones—luteolin. Flavanones—naringenin, eriocitrin. Terpenoids—menthofuran, pulegone menthol, menthone.	Antioxidant activity. Anti-inflammatory potential. Analgesic. Wound-healing effects. Immunomodulatory effects. Antibacterial activities. * Antifungal activity.	[83,228,229,230,231,232,233,234,235]
	*Rosmarinus*	*officinalis*	Diterpenes—carnosol, carnosic acid, romano, epirosmanol, 7-metylepirosmanol. Hydroxycinnamic acids—rosmarinic acid.	Antioxidant activity. Anti-inflammatory potential. Analgesic. Wound-healing effects. Immunomodulatory effects. Antibacterial activities. * Antifungal activity.	[229,236,237,238,239,240,241]
	*Leucas*	*aspera*	Flavanols—epicatechin, procyanidin. Phytosterols—*β*-sitosterol.	Antioxidant activity. Anti-inflammatory potential. Antibacterial activities. * Antifungal activity.	[242,243,244,245]
*Lythreaceae*	*Lawsonia*	*inermis*	Flavone glycosides—apigenin 5-glucoside, apigenin 7-glucoside. Hydroxybenzoic acids—gallic acid.	Antioxidant activity. Anti-inflammatory potential. Analgesic. Wound-healing effects. Immunomodulatory effects. Antibacterial activities. * Antifungal activity.	[246,247,248]
*Meliaceae*	*Azadirachta*	*indica*	Flavonols—quercetin, isoquercetin, avicularin. Ellagitannins—castalagin. Hydroxybenzoic acids—gallic acid, ellagic acid.	Antioxidant activity. Anti-inflammatory potential. Analgesic. Wound-healing effects. Immunomodulatory effects. Antibacterial activities. * Antifungal activity.	[169,249,250,251,252]
*Menispermaceae*	*Tinospora*	*cordifolia*	Diterpenes—giloin. Heteropolysaccharides—arabinogalactan. Flavonols—quercetin. Hydroxybenzoic acids—gallic acid, ellagic acid. Phenylpropanoid glycosides—syringin. Triterpenes—arjungenin, tinosporaside.	Antioxidant activity. Anti-inflammatory potential. Wound-healing effects. Immunomodulatory effects. Antibacterial activities. * Antifungal activity.	[104,131,253,254,255]
*Moraceae*	*Ficus*	*carica recemosa* *bengaalensis*	Flavonoid glycosides. Flavonols—quercetin, kaempferol. Flavonol glycosides—rutin. Hydroxybenzoic acids—gallic acid, ellagic acid, protocatechuic acid. Triterpenes—lupeol, ursolic acid, oleanolic acid. Vitamins—ascorbic acid, *α*-tocopherol.	Antioxidant activity. Anti-inflammatory potential. Analgesic. Immunomodulatory effects. Antibacterial activities. * Moderate antifungal activity.	[256,257,258,259,260,261]
*Myrtaceae*	*Melaleuca*	*alternifolia*	Monoterpenes—terpinen-4-ol. Phenylpropenes—eugenol.	Antioxidant activity. Anti-inflammatory potential. Immunomodulatory effects. Wound-healing effects. Antibacterial activities. * Antifungal activity.	[83,238,262,263,264,265,266]
	*Syzygium*	*aromaticum*	Flavonols—quercetin, kaempferol, rhamnetin. Tannins. Hydroxybenzoic acids—gallic acid, ellagic acid. Phenylpropenes—eugenol.	Antioxidant activity (low). Wound-healing effects. Analgesic. Immunomodulatory effects. Antibacterial activities. * Antifungal activity.	[83,103,267,268,269]
	*Eucalyptus*	*globulus*	Flavonol glycosides—2’’-*O*-galloylhyperin. Flavonols—quercetin, isoquercitrin. Flavanols—epicatechin.	Antioxidant activity. Anti-inflammatory potential. Wound-healing effects. Analgesic. Antibacterial activities. * Antifungal activity.	[83,270,271,272]
	*Leptospermum*	*scoparium*	Flavones—chrysin.	Antioxidant activity. Anti-inflammatory potential. Immunomodulatory effects. Wound-healing effects. Antibacterial activities. * Antifungal activity.	[67,273,274,275,276]
*Myristicaceae*	*Myristica*	*fragrans*	Monoterpenes—sabinene, *β*-pinene, *α*-pinene, carvacrol. Phenylpropenes—eugenol, myristicin, isoeugenol. Sesquiterpenes—*β*-caryophyllen.	Antioxidant activity. Anti-inflammatory potential. Analgesic. Wound-healing effects. Immunomodulatory effects. Antibacterial activities.*	[109,277,278,279,280]
*Poaceae*	*Cymbopogon*	*martini*	Flavone *C*-glycosides—isoorientin. Hydroxybenzoic acids—gallic acid. Terpenoids—cymbopogonol.	Antioxidant activity. Wound-healing effects. Antibacterial activities. Antifungal activity.	[281,282,283]
*Phyllanthaceae*	*Briedelia*	*scandens*	No data.	Antioxidant activity. Antibacterial activities. * Antifungal activity.	[284,285]
*Pinaceae*	*Cedrus*	*deodara*	Lignans—(-)-matairesinol, (-)-nortrachelogenin, dibenzylbutyrolactone lignan. Monoterpenes—linalool, *α*-terpineol, limonene. Phenylpropenes—eugenol, anethole. Sesquiterpenes—caryophyllene.	Antioxidant activity. Anti-inflammatory potential. Immunomodulatory effects. Wound-healing effects. Antibacterial activities. * Antifungal activity.	[286,287,288,289,290]
*Rutaceae*	*Aegle*	*marmelos*	Flavonoid glycosides. Flavonols—quercetin. Flavonol glycosides—rutin. Hydroxycinnamic acids—coumaric acid, caffeic acid, *p*-coumaric acid, chlorogenic acid. Hydroxybenzoic acids—vanillic acid. Vitamins—ascorbic acid, α-tocopherol.	Antioxidant activity. Anti-inflammatory potential. Analgesic. Immunomodulatory effects. Wound-healing effects. Antibacterial activities. * Antifungal activity.	[105,130,291,292,293,294]
	*Citrus*	*limon*	Carotenoids—*β*-carotene, zeaxanthin, lutein, lycopene. Flavanones—naringenin, hesperidin. Flavones—tangeritin. Flavonols—quercetin. Flavonol glycosides—rutin. Vitamins—ascorbic acid.	Antioxidant activity. Anti-inflammatory potential. Analgesic. Immunomodulatory effects. Wound-healing effects. Antibacterial activities * (except for *S. aureus*). No antifungal activity.	[295,296,297,298]
*Nyctaginaceae*	*Boerhaavia*	*diffusa*	Flavonols—quercetin, kaempferol. Phenolic compounds—punarnavoside. Rotenoids—boeravinone G.	Antioxidant activity. Anti-inflammatory potential. Analgesic. Immunomodulatory effects. Wound-healing effects. Antibacterial activities. * Antifungal activity.	[299,300,301,302,303,304]
*Salvadoraceae*	*Salvadora*	*persica*	Flavonoids. Hydroxybenzoic acids—gallic acid. Hydroxycinnamic acids—caffeic acid. Sterols—*β*-sitosterol. Tannins.	Antioxidant activity. Anti-inflammatory potential. Analgesic. Wound-healing effects. Antibacterial activities. * Antifungal activity.	[305,306,307]
*Sapindaceae*	*Schleichera*	*oleosa*	Flavanols—catechin, epicatechin. Flavonols—kaempferol. Hydroxybenzoic acids—gallic acid, ellagic acid.	Antioxidant activity. Anti-inflammatory potential. Analgesic. Wound-healing effects. Antibacterial activities.	[94,308,309,310,311]
*Simaroubaceae*	*Ailanthus*	*altissima*	Hydroxycinnamic acids—ferulic acid. Flavanols—catechin. Flavonols—quercetin. Flavonol glycosides—rutin.	Antioxidant activity. Anti-inflammatory potential. Analgesic. Wound-healing effects. Antibacterial activities. *	[312,313,314,315]
*Solanaceae*	*Solanum*	*nigrum*	Flavanones—naringenin. Flavonols—quercetin, isoquercitrin. Flavonol glycosides—rutin. Hydroxybenzoic acids—gallic acid, protocatechuic acid.	Antioxidant activity. Anti-inflammatory potential. Analgesic. Immunomodulatory effects. Antibacterial activities. * Antifungal activity.	[316,317,318,319,320]
	*Datura*	*stramonium*	Coumarins—scopoletin. Flavonols—quercetin. Hydroxybenzoic acids—gallic acid. Steroidal lactones—daturametelin B, daturamalakoside B.	Antioxidant activity. Anti-inflammatory potential. Analgesic. Immunomodulatory effects. Wound-healing effects. Antibacterial activities.	[321,322,323]
	*Capsicum*	*annuum*	Flavones—luteolin. Flavanols—catechin, epicatechin. Flavonol glycosides—rutin. Hydroxybenzoic acids—gallic acid. Stilbenes—resveratrol. Vitamins—ascorbic acid.	Antioxidant activity. Anti-inflammatory potential. Analgesic. Antibacterial activities. *	[324,325,326,327,328]
*Umbelaceae*	*Pimpinella*	*anisum*	Esters of gallic acid—methyl gallate. Flavanones—naringenin, hesperetin. Flavanols—catechin. Flavonols—quercetin. Flavonol glycosides—rutin. Hydroxybenzoic acids—gallic acid. Hydroxycinnamic acids—cinnamic acid, caffeic acid. Isoflavones—daidzein. Phenylpropenes—eugenol, anethole, estragole.	Antioxidant activity. Anti-inflammatory potential. Wound-healing effects. Antibacterial activities. * Antifungal activity.	[329,330,331,332]
*Verbanaceae*	*Lippia*	*multiflora*	Phenylethanoid glycosides—verbascoside, isoverbascoside, nuomioside A, isonuomioside A.	Antioxidant activity. Anti-inflammatory potential. Analgesic. Antibacterial activities. *	[333,334,335]
*Lamiaceae*	*Vitex*	*negundo*	Hydroxycinnamic acids—chlorogenic acid. Flavone *C*-glycosides—isoorientin. Flavones—cynaroside, scutellarin, vitexin. Phenolic compounds—vitedoin A, vitexnegheteroins. *p*-hydroxybenzoic acid derivatives—agnuside.	Antioxidant activity. Anti-inflammatory potential. Analgesic. Immunomodulatory effects. Wound-healing effects. Antibacterial activities. * Antifungal activity.	[336,337,338,339,340]
	*Clerodendrum*	*infortunatum*	Diterpene alcohols—phytol. Fatty acids—hexadecanoic acid. Hydroxybenzoic acids—gallic acid. Phytosterols—stigmasterol. Terpenoids—oleanolic acid, clerodinin A.	Antioxidant activity. Anti-inflammatory potential. Analgesic. Wound-healing effects. Antibacterial activities. Antifungal activity.	[341,342,343,344]
*Zingiberaceae*	*Curcuma*	*longa*	Curcuminoids—curcumin, demethoxycurcumin, bisdemethoxycurcumin. Hydroxycinnamic acids—caffeic acid. Flavonols—quercetin, isorhamnetin.	Antioxidant activity. Anti-inflammatory potential. Analgesic. Wound-healing effects. Antibacterial activities. * Antifungal activity.	[83,345,346,347,348]

Antibacterial activities *—the antibacterial activity of the raw material covers the strains of *S. aureus.*

## 5. Antioxidant Properties of Medicinal Plants in Treating Scabies

The relationship between biologically active compounds in plant extracts and oxidative stress is intricate and not yet fully understood. Clarifying these interactions is essential for optimizing therapeutic doses and enhancing the efficacy of plant-based treatments, such as antiscabies therapies. Direct application of medicinal plant extracts or oils to the skin can offer a natural remedy for scabies. For instance, lemon oil contains D-limonene, a compound that disrupts the respiratory system of insects and mites, demonstrating its potential acaricidal properties. A study by Aboelhadid et al. [349] found that application of 20% lemon oil significantly increased lipid peroxide levels in mites, indicating oxidative stress. The study found higher levels of hydrogen peroxide and malondialdehyde in mites treated with 20% lemon oil compared to those treated with deltamethrin or distilled water. Furthermore, lemon oil at concentrations of 10% and 20% caused 100% mite mortality within 24 h. Interestingly, increasing the concentration of lemon oil to 50% or 100% did not result in proportionally higher oxidative stress. This observation suggests a nonlinear, potentially asymptotic dose-response relationship, where the efficacy of lemon oil reaches a maximum threshold above which no further benefit is observed, possibly due to the manifestation of toxic effects of certain constituents within the complex plant extract at higher concentrations. Recognizing these nonlinear, curvilinear, and asymptotic dose-response relationships is essential to understanding the complex interactions between plant extracts and biological systems. Future studies should aim to precisely quantify these relationships in order to optimize dosing strategies for plant-based therapies, maximizing their therapeutic benefits while minimizing unnecessary exposure and potential side effects.

The potent antioxidant activity of *Salvadora persica* is primarily attributed to its high content of flavonoids and phenolic compounds, which neutralize free radicals by donating electrons and thereby preventing oxidative damage to key cellular structures such as lipids, proteins, and DNA. Additionally, sulfur-containing compounds in *S. persica* further enhance its antioxidant capacity, contributing to its effectiveness in mitigating oxidative stress and inflammation [305]. Notably, bioactive compounds like benzyl nitrile and isothiocyanatomethyl in *S. persica* exhibit strong binding affinity with the scabies protease paralogues SMIPP-S-D1, indicating their potential as effective treatments for scabies. Both methanol and ethanol extracts of *S. persica* have demonstrated significant free radical scavenging activity, with the methanol extract achieving a scavenging rate of up to 96%, and the ethanol extract up to 95%. These findings suggest that *S. persica* extracts are powerful antioxidants, comparable to or even surpassing standard antioxidants like ascorbic acid, which showed an 89% scavenging rate. This antioxidant activity plays a crucial role in protecting skin cells from oxidative damage during scabies infestation and promotes overall skin healing [305]. Future studies should focus on optimizing the use of *S. persica* extracts to fully exploit their therapeutic potential, investigating dose-response relationships, and assessing long-term safety and efficacy in clinical settings.

**Figure 1:**
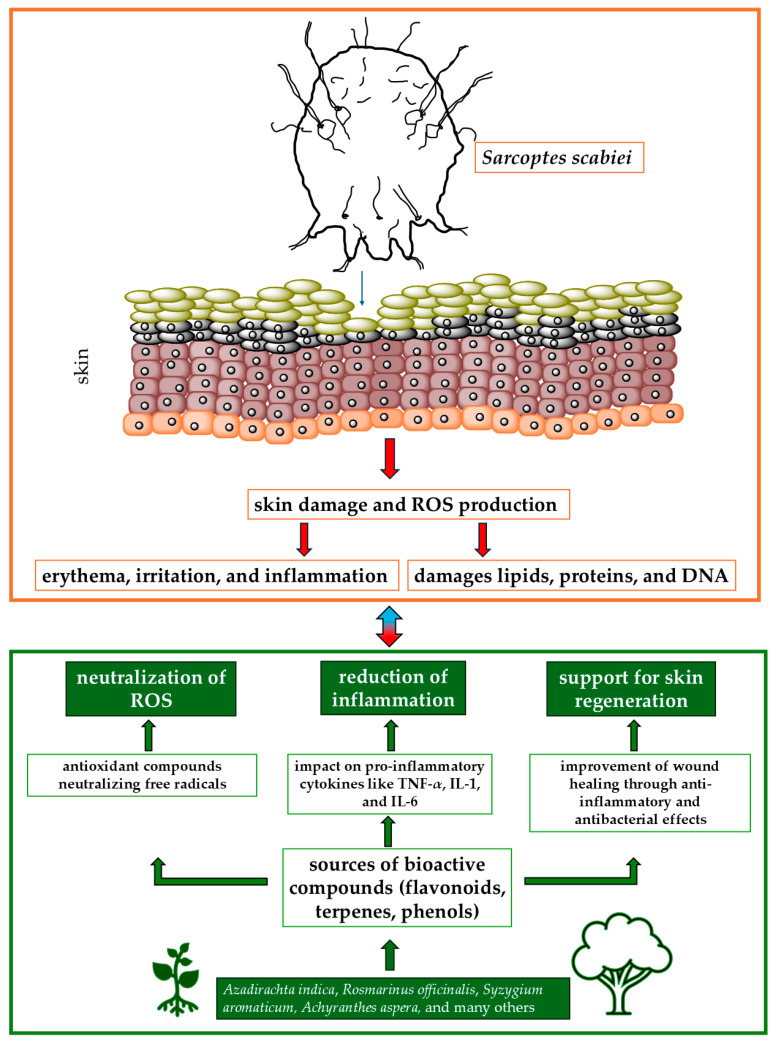
Mechanisms of action of medicinal plants in treating scabies and reducing oxidative stress. Abbreviations: IL-1, IL-6: interleukin 1 or 6; ROS: reactive oxygen species; TNF-α; tumor necrosis factor-α.

The available literature on medicinal plants with antiscabies and antioxidant properties highlights their promising potential in the treatment of scabies. Their antioxidant activity not only combats oxidative stress but also enhances the overall therapeutic effect against scabies. For example, Danino et al. [81] tested biologically active compounds isolated from the methanolic extract of *Inula viscosa*, a plant selected for its potential in the treatment of skin diseases such as scabies. The extract contains polyphenolic antioxidants, including 1,3-dicaffeoylquinic acid, which belongs to the caffeoylquinic acid family. The antioxidant activity of 1,3-dicaffeoylquinic acid does not increase proportionally with the dose, showing a nonlinear and probably asymptotic relationship, which is crucial for optimizing its therapeutic use. Similarly, *Scabiosa columbaria*, a plant commonly used in traditional medicine for the treatment of scabies, has shown significant antioxidant properties. These properties are largely attributed to the presence of various phenolic compounds, such as chlorogenic acid, caffeic acid, and catechin, which are abundant in both the leaves and flowers of the plant. The antioxidant activity of *S. columbaria* is particularly strong in the leaves, where the phenolic content is higher. The strong antioxidant properties of *S. columbaria*, resulting from its rich content of phenolic compounds and essential oils, make it a promising candidate for the treatment of scabies. Its ability to reduce oxidative stress and promote the healing of skin lesions could potentially increase its efficacy in traditional medicinal applications against scabies. Figure 1 shows the relationships between active compounds obtained from plants and the effects of *S. scabiei*.

## 6. Conclusions and Future Perspectives

Medicinal plants with antioxidant properties present a promising alternative for the treatment of scabies, particularly due to their capacity to neutralize free radicals and mitigate oxidative stress. These plants offer multiple therapeutic benefits, including accelerated skin healing, reduction in inflammation, and antibacterial effects, which are essential for alleviating the symptoms of scabies. However, challenges such as variability in plant composition, standardization of extracts, and the lack of rigorous clinical testing must be addressed to fully integrate these natural remedies into clinical practice. As resistance to conventional scabicides continues to rise, the importance of exploring alternative treatments becomes increasingly critical.

Plant extracts like lemon oil and *Salvadora persica* have demonstrated significant antioxidant and antiscabies potential, making them viable alternatives to conventional acaricides. In addition to combating oxidative stress, these extracts enhance the overall therapeutic effects against scabies. Nevertheless, further research is required to define the precise dose-response relationship, optimal concentrations, and long-term efficacy of these natural treatments. Future studies should focus on clinical trials to validate the effectiveness of medicinal plants, their safety profiles, and potential integration into standard scabies treatment protocols. As resistance to chemical treatments grows, medicinal plants may offer a sustainable and effective approach in scabies management.

## Data Availability

Not applicable.
